# An asymptotic approximation to the cable equation for arbitrary diameter taper

**DOI:** 10.1186/1471-2202-16-S1-P40

**Published:** 2015-12-18

**Authors:** Alex D Bird, Hermann Cuntz

**Affiliations:** 1 Warwick Systems Biology Centre, University of Warwick, Coventry, CV4 7AL, UK; 2 School of Life Sciences, University of Warwick, Coventry, CV4 7AL, UK; 3 Warwick Systems Biology DTC, University of Warwick, Coventry, CV4 7AL, UK; 4 Ernst Strüngmann Institute for Neuroscience, Frankfurt am Main, Germany; 5 Frankfurt Institute for Advanced Studies, Frankfurt am Main, Germany

## 

Somatic integration of synaptic inputs relies on the propagation of currents arising from sources across the dendritic tree. Whilst active processes strongly contribute to current flow in most neurons, understanding the passive backbone to transmission allows a better intuitive grasp of dendritic function; the results of Rall in highlighting the properties of cylindrical dendrites[1] are of foundational importance in compartmental modelling and computational neuroscience. Dendrites are, however, not generally cylindrical, they tend to taper in a way that contributes to the normalisation of input currents towards the soma[2].

**Figure 1:**
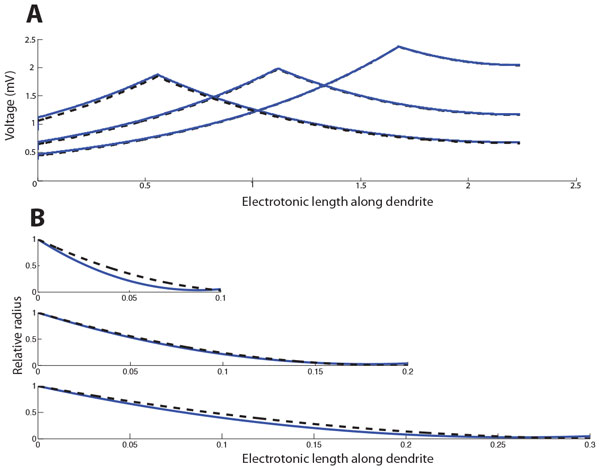
**Asymptotic approximations reveal fundamental properties of voltage flow in tapering dendrites**. **A **Comparison of numerical (blue) and first-order asymptotic (black dashed) methods for determining the steady-state voltage in a quadratically tapering dendritic cable for currents injected at three different sites. **B **Comparison of numerically-optimised tapering profiles (blue) with those predicted by the asymptotic approximation (black dashed) for dendrites of different electrotonic length.

We have derived an asymptotic approximation to the voltage in dendrites with an arbitrary taper profile using the insight that voltage attenuation is substantially faster than radius change in realistic morphologies (Figure 1A). This result allows faster computation and greater insight than standard approaches using large numbers of cylinders or frusta to numerically compute voltage profiles. In addition, it provides easy generalisations of the standard results of cable theory involving transients and branches.

A particularly interesting implication of these results is that the optimal taper profile to proximally transmit maximum voltages can be found by variational calculus, matching results from non-parametric numerical optimisation which predicted a quadratic form for the diameter taper (Figure 1B) [3].
